# Four-octyl itaconate activates Nrf2 cascade to protect osteoblasts from hydrogen peroxide-induced oxidative injury

**DOI:** 10.1038/s41419-020-02987-9

**Published:** 2020-09-17

**Authors:** Yuehuan Zheng, Zhe Chen, Chang She, Yazhou Lin, Yuan Hong, Liqiang Shi, Yingzi Zhang, Peng Cao, Xiangyang Xu

**Affiliations:** 1 Department of Orthopedics, Ruijin Hospital, Shanghai Jiao Tong University School of Medicine, Shanghai, China; 2 Department of Orthopedics, Ruijin Hospital North, Shanghai Jiao Tong University School of Medicine, Shanghai, China; 3 Department of Orthopedics, The Second Affiliated Hospital of Soochow University, Suzhou, China

**Keywords:** Apoptosis, Stress signalling

## Abstract

Four-octyl itaconate (4-OI) is the cell-permeable derivative of itaconate that can activate Nrf2 signaling by alkylating Keap1’s cysteine residues. Here, we tested the potential effect of 4-OI on hydrogen peroxide (H_2_O_2_)-induced oxidative injury in osteoblasts. In OB-6 cells and primary murine osteoblasts, 4-OI was able to activate Nrf2 signaling cascade and cause Keap1–Nrf2 disassociation, Nrf2 protein stabilization, cytosol accumulation, and nuclear translocation. 4-OI also augmented antioxidant-response element reporter activity and promoted expression of Nrf2-dependent genes (*HO1*, *NQO1*, and *GCLC*). Pretreatment with 4-OI inhibited H_2_O_2_-induced reactive oxygen species production, cell death, and apoptosis in osteoblasts. Furthermore, 4-OI inhibited H_2_O_2_-induced programmed necrosis by suppressing mitochondrial depolarization, mitochondrial cyclophilin D-ANT1 (adenine nucleotide translocase 1)-p53 association, and cytosol lactate dehydrogenase release in osteoblasts. Ectopic overexpression of immunoresponsive gene 1 (IRG1) increased endogenous itaconate production and activated Nrf2 signaling cascade, thereby inhibiting H_2_O_2_-induced oxidative injury and cell death. In OB-6 cells, Nrf2 silencing or CRISPR/Cas9-induced Nrf2 knockout blocked 4-OI-induced osteoblast cytoprotection against H_2_O_2_. Conversely, forced Nrf2 activation, by CRISPR/Cas9-induced Keap1 knockout, mimicked 4-OI-induced actions in OB-6 cells. Importantly, 4-OI was ineffective against H_2_O_2_ in Keap1-knockout cells. Collectively, 4-OI efficiently activates Nrf2 signaling to inhibit H_2_O_2_-induced oxidative injury and death of osteoblasts.

## Introduction

Sustained reactive oxygen species (ROS) production will induce profound oxidative injury to human osteoblasts^1^, and it is one of the primary pathology of osteoporosis and/or osteonecrosis^2,3^. Adding hydrogen peroxide (H_2_O_2_) to cultured osteoblastic cells or primary osteoblasts is an established cellular model of osteoporosis/osteonecrosis. H_2_O_2_ will induce significant oxidative stress, protein damage, and DNA breaks that eventually lead to cell apoptosis and necrosis^4–8^. This model has been utilized to understand the mechanisms of H_2_O_2_-induced osteoblast cell injury and to explore possible intervention strategies^4–8^.

Nuclear factor E2-related factor 2 (Nrf2) is a well-defined signaling cascade that offers significant cellular protection against various oxidative stimuli^9^. Forced activation of Nrf2 cascade through genetic strategies or pharmacological agents can protect osteoblasts from H_2_O_2_ and other oxidative injury^4,6,8,10,11^. Wang et al. have shown that MIND4-17 was able to activate Nrf2 signaling cascade to protect OB-6 human osteoblastic cells and primary osteoblasts from H_2_O_2_^8^. Han et al. demonstrated that chlorogenic acid could activate PI3K-Akt-dependent Nrf2 signaling and inhibit H_2_O_2_-induced oxidative injury in MC3T3-E1 osteoblastic cells^10^. MicroRNA-455 (miR-455), which silenced Cullin 3, protected hFOB1 19 osteoblast cells and primary human osteoblasts from H_2_O_2_ by causing Nrf2 protein stabilization and Nrf2 cascade activation^6^. Therefore, the activation of Nrf2 cascade is a fine strategy to protect osteoblasts/osteoblastic cells from H_2_O_2_.

In the unstimulated resting condition, Nrf2 localizes in the cytoplasm and binds to Keap1 (Kelch-like ECH-associated protein 1)^12,13^, and the latter dictates Nrf2 proteasome degradation through Cullin 3 ubiquitin complex. Once activated Nrf2 departs from Keap1, Nrf2 protein would undergo stabilization and cytosol accumulation. Stabilized Nrf2 protein translocates to the cell nuclei and binds to antioxidant-response elements (ARE), which then promotes transcription and mRNA expression of antioxidant genes and detoxifying enzymes^12,13^. Nrf2-ARE-dependent genes include *heme oxygenase 1* (*HO1*), *γ-glutamyl cysteine ligase catalytic subunit* (*GCLC*), *NAD(P)H quinone oxidoreductase-1* (*NQO1*), *glutathione (GSH)*, and many others. All of them exert significant antioxidant and cytoprotective functions^12,13^.

Recent studies have discovered itaconate as a novel Nrf2 activator^14,15^. Itaconate alkylates Keap1’s key cysteine residues to activate Nrf2 signaling cascade by causing Nrf2-Keap1 disassociation and Nrf2 protein stabilization^14^. A cell-permeable itaconate derivative, 4-octyl itaconate (4-OI), was synthesized and it efficiently activated Nrf2 signaling in mammalian cells^14,16–18^. The potential activity of 4-OI in osteoblasts has not been studied fully. Here, we reported that the activation of Nrf2 cascade by 4-OI was able to protect osteoblasts from H_2_O_2_-induced oxidative injury and cell death.

## Materials and methods

### Reagents, chemicals, and antibodies

Four-octyl itaconate (4-OI) was synthesized by Ruilu Chemicals (Shanghai, China). H_2_O_2_ was provided by Sigma Aldrich Chemicals (St Louis, Mo). Antibodies for HO1 (#70081), NQO1 (#3187), Nrf2 (#12721), Keap1 (#8047), Tubulin (#2125), and Lamin B1 (#13435), as well as cleaved-poly (ADP-ribose) polymerase (PARP, #5625), cleaved-caspase-9 (#20750), and immunoresponsive gene 1 (IRG1, #77510) were obtained from Cell Signaling Tech China (Shanghai, China). The anti-GCLC antibody (ab55435) and the anti-adenine nucleotide translocase-1 (ANT1) antibody (ab102032) were purchased from Abcam China (Shanghai, China). Antibodies of PGK1 (sc-130335) and cyclophilin D (CyPD, sc-137136) were provided by Santa Cruz Biotech (Santa Cruz, CA). RNA assay reagents were obtained from Thermo-Fisher Invitrogen (Suzhou, China). Cell Counting Kit-8 (CCK-8) was provided by Dojindo Laboratories (Kumamoto, Japan).

### Cell culture

The differentiated OB-6 human osteoblastic cells and the primary murine osteoblasts were provided by Dr. Ji^19,20^. 4-OI was dissolved in DMSO in the stock solution. To test its cytoprotective activity, OB-6 osteoblastic cells or primary murine osteoblasts were pretreated with 4-OI (with final DMSO concentration at 0.2%) for 2 h, followed by H_2_O_2_ stimulation. Cells were routinely subjected to mycoplasma and microbial contamination examination every 2–3 months. STR profiling, population doubling time, and morphology were regularly checked to confirm the genotype. The protocols were approved by the Ethics Committee of Ruijin Hospital, Shanghai Jiao Tong University School of Medicine, according to the principles of Declaration of Helsinki.

### Quantitative real-time PCR (qPCR)

Following treatment, the total cellular RNA was extracted by TRIzol reagents^21^. The qPCR procedures using a SYBR Green PCR kit (Applied Biosystems, Suzhou, China) under the ABI Prism7500 Fast Real-Time PCR system were reported before^22^. To calculate the product melting temperature, the melting curve analysis was carried out. Glyceraldehyde-3-phosphate dehydrogenase (*GAPDH*) was examined as the internal control and reference gene. Quantification was through the 2^−∆∆*C*t^ method. The mRNA primers for *Nrf2*, *HO1*, *NQO1*, *GCLC*, and *GAPDH* were provided by Dr. Di^18^. The primers of *immunoresponsive gene 1* (*IRG1*) were purchased from OriGene (Beijing, China).

### Antioxidant-response element (ARE) reporter activity

Osteoblastic cells/osteoblasts were seeded into six-well plates and transfected with an ARE-inducible firefly luciferase vector (provided by Dr. Jiang^22^). Following the applied treatments, ARE firefly luciferase activity was tested by the luminescence.

### Western blotting

The detailed protocols for western blotting were described before^22^. An ImageJ software (from NIH) was utilized for data quantification. The same set of lysates were run in parallel (“sister”) gels. To separate cell nuclei, a nuclei isolation kit (from Sigma) was applied^23^.

### Co-immunoprecipitation (Co-IP)

A total of 700 μg of protein lysates from OB-6 cells were pre-cleared with A/G beads (Sigma). The lysates were then incubated with anti-Keap1 antibody overnight^11^. The Keap1-bound proteins were captured by protein IgA/G beads, and examined by western blotting.

### Mitochondrial immunoprecipitation (Mito-IP)

OB-6 cells were seeded into six-well tissue-culture plates. The detailed protocols of isolating mitochondrial fraction lysates and mito-IP were described before^24^.

### Alkaline phosphatase (ALP) assay

The primary murine osteoblasts were seeded into 12-well plates (6 × 10^4^ cells per well). Murine osteoblasts were stained with ALP using an ALP staining Kit (Beyotime Institute of Biotechnology, Wuxi, China) and cultured for 12 days. The representative ALP staining image is shown.

### Cell viability

OB-6 osteoblastic cells or primary murine osteoblasts were seeded into 96-well plates (at 2 × 10^3^ cells per well). Following treatment, a CCK-8 assay kit was utilized to quantify cell viability, with CCK-8’s optical density (OD) examined at 490 nm.

### Cell apoptosis-related assays

Cell apoptosis-related assays, including the caspase-3 activity assay, Annexin V-propidium iodide (PI) FACS, and nuclear terminal deoxynucleotidyl transferase dUTP nick-end labeling (TUNEL) staining were described in other studies^23,25,26^. For TUNEL staining, TUNEL/DAPI ratio was calculated from recording at least 500 cells from five random microscope views (1 × 100).

### Mitochondrial depolarization

With cells undergoing mitochondrial depolarization, the JC-1 fluorescence dye (Sigma) accumulates in the mitochondria to form green monomers^27^. The detailed protocols for JC-1 assay of mitochondrial depolarization have been described before^11^. The JC-1 fluorescence images with merging green and red fluorescence channels are presented.

### ROS detection

OB-6 osteoblastic cells or primary murine osteoblasts were seeded into 12-well tissue-culture plates. After treatment, cells were stained with CellROX (5 μM, Invitrogen-Thermo Fisher). CellROX intensity was tested by a fluorescent spectrophotometer. CellROX images were represented.

### Glutathione contents

OB-6 cells or primary murine osteoblasts were seeded into six-well plates. The ratio of reduced glutathione (GSH) to the oxidized disulfide form glutathione (GSSG)^28^ was tested using a previously described protocol^28^.

### Single-strand DNA (ssDNA) ELISA

OB-6 cells or primary murine osteoblasts were seeded into six-well plates. Following treatment, the cellular ssDNA contents were examined by an ApoStrandTM ELISA kit (BIOMOL International, Plymouth Meeting, PA). The ssDNA ELISA OD was recorded at 450 nm.

### Lactate dehydrogenase (LDH) assay

OB-6 cells or primary murine osteoblasts were seeded into 12-well tissue-culture plates (6 × 10^4^ cells per well). Following treatment, a two-step simple LDH assay kit (Takara, Tokyo, Japan) was utilized to quantify LDH contents in the medium, which were then normalized to total LDH contents.

### Nrf2 short-hairpin RNA (shRNA)

OB-6 cells were seeded into six-well plates and transfected with the Nrf2 shRNA lentiviral particles (sc-37030V, Santa Cruz Biotech, Santa Cruz, CA). To select stable cells, puromycin (2.5 μg/mL) was added in the complete medium (for 4–5 passages). Nrf2 silencing in the stable cells, namely, sh-Nrf2 cells, was confirmed by western blotting and qPCR analyses.

### CRISPR-Cas9-mediated gene knockout (KO)

OB-6 cells were transduced with a CRISPR/Cas9-Nrf2-KO-GFP-puro construct or a CRISPR/Cas9-Keap1-KO-GFP-puro construct provided by Dr. Liu^18^. The GFP-positive transfected cells were sorted by FACS. The selected cells were distributed into 96-well plates and cultured in puromycin (2.5 μg/mL)-containing medium. In stable cells, Nrf2 KO or Keap1 KO was verified by western blotting and qPCR analyses.

### Forced expression of IRG1

The full-length human *IRG1 cDNA* (provided by Genechem) was sub-cloned into a GV369 lentiviral construct (Genechem, Shanghai, China). The construct was transfected to HEK-293 cells together with the lentivirus-packing helper plasmids (Genechem) to generate the *IRG1*-expressing lentivirus. The lentivirus was added to OB-6 cells (cultured in polybrene-containing complete medium). Following selection by puromycin (2.5 μg/ml, for 5–6 passages), stable cells were established: OE-IRG1 cells. IRG1 overexpression was verified by western blotting and qPCR analyses.

### Itaconate contents

OB-6 cells were seeded into six-well tissue-culture plates. The cellular itaconate contents were tested by a commercial kit from Biyuntian (Wuxi, China) using the attached protocol. The levels were normalized to control.

### Statistical analysis

The investigators were blinded to group allocation. Quantitative data with normal distribution were always presented as mean ± standard deviation (SD). Statistical analyses were performed by two-way ANOVA using a Scheffe’s *F* test (SPSS 23.0, SPSS Co.. Chicago, IL). To test significance between two treatment groups, a two-tailed unpaired *T* test (Excel 2007) was utilized. *P* < 0.05 was considered as statistically significant.

## Results

### Nrf2 cascade activation by 4-OI in osteoblasts

**Fig. 1:**
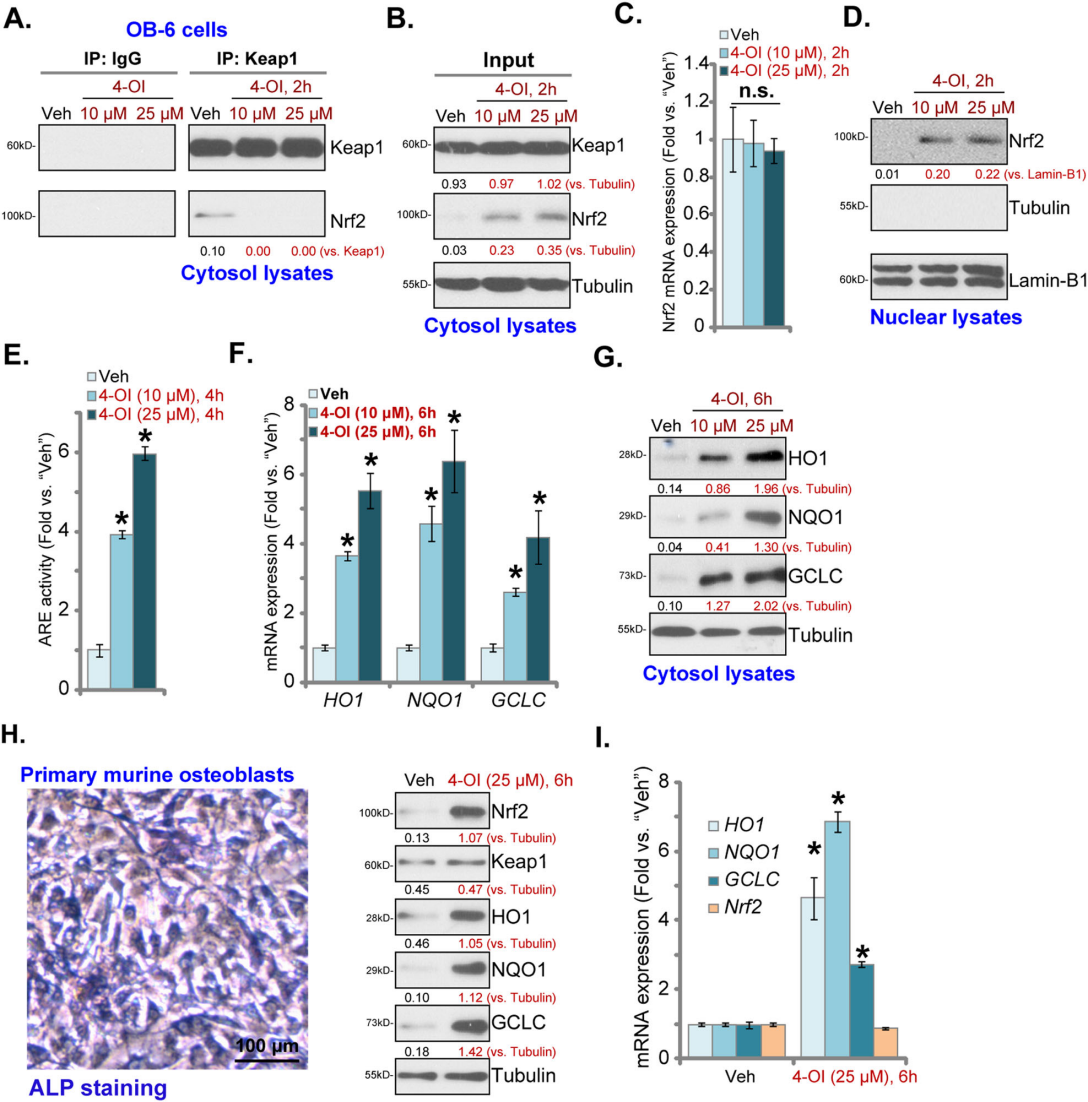
Nuclear factor E2-related factor 2 (Nrf2) cascade activation by 4-OI in osteoblasts. Human osteoblastic OB-6 cells (**a**–**g**) or the primary murine osteoblasts (**h**, **i**) were treated with 4-OI (10/25 μM) or vehicle control (0.2% of DMSO, “Veh”) and cultured for applied time periods; Keap1–Nrf2 association was tested by co-immunoprecipitation (Co-IP assay) (**a**). Expression of listed proteins in cytosol fraction lysates and nuclei fraction lysates was tested by western blotting (**b**, **d**, **g**, **h**), with the expression of listed mRNAs tested by qPCR (**c**, **f**, **i**). The relative ARE reporter luciferase activity was tested as well (**e**). The morphology of murine osteoblasts with ALP staining is shown (**h**, the left panel). Expression of the listed proteins was quantified and normalized to the loading control (**a**, **b**, **d**, **g**, **h**). Quantified values were mean ± standard deviation (SD, *n* = 5). “n.s.” stands for no statistical difference (**c**). *P < 0.05 vs. “Veh” treatment. Experiments were repeated three times, with similar results obtained. Bar = 100 μm (**h**).

We first tested whether 4-OI can provoke Nrf2 signaling cascade in human osteoblasts. The co-immunoprecipitation (Co-IP) assay results in Fig. 1a demonstrated that Keap1 immunoprecipitated with Nrf2 in the vehicle (0.2% of DMSO)-treated control OB-6 human osteoblastic cells^8,29^. The Keap1–Nrf2 association was disrupted with stimulation of 4-OI (Fig. 1a). 4-OI was utilized at 10–25 μM, referring other studies^14,16,18^. Following disassociation with Keap1, the Nrf2 protein was stabilized and accumulated in 4-OI-treated OB-6 cells (Fig. 1b). Keap1 protein expression was unchanged (Fig. 1b). Nrf2 protein elevation by 4-OI was unlikely due to de novo protein synthesis since *Nrf2* mRNA levels were not significantly increased (Fig. 1c). As Nrf2 protein levels were dramatically increased in the nuclei fraction lysates of 4-OI-treated OB-6 cells, stabilized Nrf2 protein translocated to cell nuclei (Fig. 1d). Importantly, the relative reporter activity of ARE was robustly elevated following 4-OI treatment in OB-6 cells (Fig. 1e), resulting in the increased transcription and mRNA expression of Nrf2-ARE-dependent genes: *HO1*, *NQO1*, and *GCLC* (Fig. 1f). Protein expressions of HO1, NQO1, and GCLC were significantly upregulated as well in 4-OI-treated OB-6 cells (Fig. 1g). These results clearly showed that 4-OI is able to activate Nrf2 signaling cascade in OB-6 cells. Notably, 4-OI displayed a concentration-dependent activity as it was more efficient in activating Nrf2 cascade at 25 μM than at 10 μM (Fig. 1a–g).

In the primary murine osteoblasts (the morphology with ALP staining shown in Fig. 1h), treatment with 4-OI (25 μM) activated Nrf2 cascade, leading to Nrf2 protein stabilization (Fig. 1h), and increased mRNA and protein expression of Nrf2-ARE-dependent genes, *HO1*, *NQO1*, and *GCLC* (Fig. 1h, i).

### H_2_O_2_-induced oxidative injury is inhibited by 4-OI in osteoblasts

**Fig. 2:**
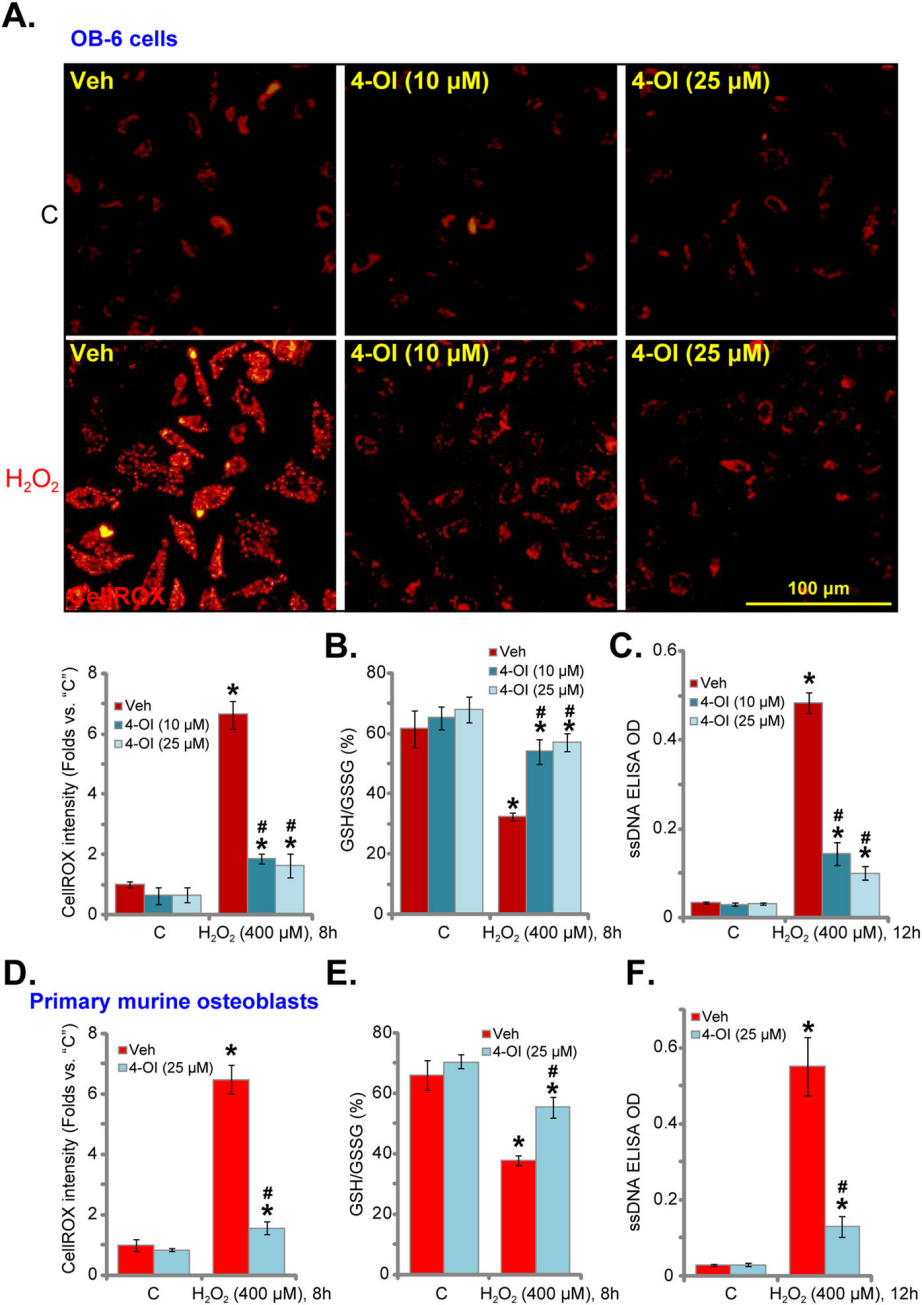
H_2_O_2_-induced oxidative injury was inhibited by 4-OI in osteoblasts. Human osteoblastic OB-6 cells (**a**–**c**) or primary murine osteoblasts (**d**–**f**) were pretreated for 2 h with 4-OI (10/25 μM) or vehicle control (“Veh”), followed by H_2_O_2_ (400 μM) stimulation. Cells were further cultured for applied time periods, reactive oxygen species (ROS) production (tested by CellROX intensity, **a**, **d**), the GSH/GSSG ratio (**b**, **e**), and single-strand DNA (ssDNA) contents (ELISA OD, **c**, **f**) were tested. Quantified values were mean ± standard deviation (SD, *n* = 5). “C” stands for the untreated control cells. *P < 0.05 vs. “C” cells. ^#^*P* < 0.05 vs. cells with H_2_O_2_ stimulation but “Veh” pretreatment. Experiments were repeated three times, with similar results obtained. Bar = 100 μm (**a**, **d**).

H_2_O_2_ stimulation in cultured osteoblasts/osteoblastic cells would induce oxidative stress and cause profound protein damage, lipid peroxidation, and DNA breaks^4,8,30,31^. In human osteoblastic OB-6 cells, H_2_O_2_ (400 μM) significantly increased ROS production, evidenced by the increased CellROX intensity^32,33^ (Fig. 2a). Further confirming the oxidative injury, the GSH/GSSG ratio was decreased following H_2_O_2_ stimulation in OB-6 cells (Fig. 2b). Additionally, levels of single-strand DNA (ssDNA) were increased in H_2_O_2_-treated OB-6 cells (Fig. 2c). Significantly, pretreatment with 4-OI (at 10/25 μM) largely inhibited H_2_O_2_-induced oxidative injury in OB-6 cells, and suppressed CellROX intensity increase (Fig. 2a), GSH/GSSG ratio reduction (Fig. 2b), and ssDNA accumulation (Fig. 2c).

Similar results were detected in primary murine osteoblasts as well, since 4-OI (25 μM) pretreatment significantly attenuated H_2_O_2_-induced ROS production (CellROX intensity increase, Fig. 2d), the GSH/GSSG ratio reduction (Fig. 2e), and ssDNA accumulation (Fig. 2f). Importantly, 4-OI single treatment failed to alter basal oxidative levels in OB-6 cells (Fig. 2a–c) and primary murine osteoblasts (Fig. 2d–f).

### H_2_O_2_-induced apoptosis is inhibited by 4-OI in osteoblasts

**Fig. 3:**
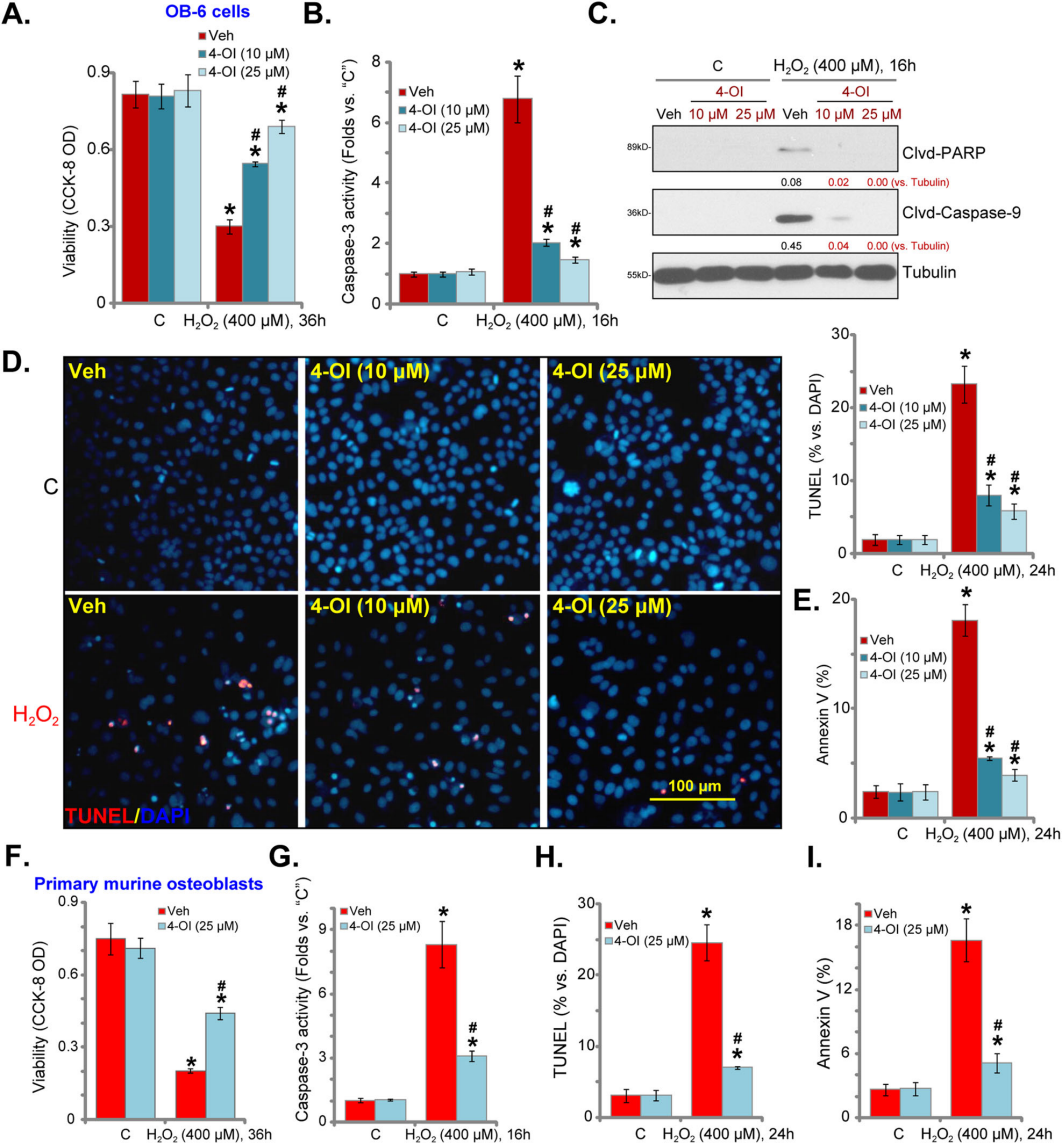
H_2_O_2_-induced apoptosis is inhibited by 4-OI in osteoblasts. Human osteoblastic OB-6 cells (**a**–**e**) or primary murine osteoblasts (**f**–**i**) were pretreated for 2 h with 4-OI (10/25 μM) or vehicle control (“Veh”), followed by H_2_O_2_ (400 μM) stimulation. Cells were further cultured for applied time periods; cell viability (CCK-8 OD, **a**, **f**), caspase-3 activity (**b**, **g**), and expression of listed proteins (western blotting assays, **c**) were tested; cell apoptosis was examined by nuclear TUNEL staining (**d**, **h**) and Annexin V FACS (**e**, **i**) assays, data were quantified. Expression of the listed proteins was quantified and normalized to the loading control (**c**). Quantified values were mean ± standard deviation (SD, *n* = 5). “C” stands for the untreated control cells. *P < 0.05 vs. “C” cells. ^#^*P* < 0.05 vs. cells with H_2_O_2_ stimulation but “Veh” pretreatment. Experiments were repeated three times, with similar results obtained. Bar = 100 μm (**d**).

Next, we studied whether 4-OI can inhibit H_2_O_2_-induced cytotoxicity in osteoblasts. CCK-8 viability assay results demonstrated that in OB-6 osteoblastic cells, H_2_O_2_ (400 μM) treatment potently decreased CCK-8 OD (Fig. 3a). This was largely attenuated by pretreatment of 4-OI (10/25 μM) (Fig. 3a). H_2_O_2_-induced caspase-3 activation (Fig. 3b) as well as cleavages of caspase-9 and poly (ADP-ribose) polymerase (PARP) (Fig. 3c) were both inhibited with 4-OI pretreatment. H_2_O_2_ provoked apoptosis activation, increased TUNEL-positive nuclei ratio (Fig. 3d), and the number of Annexin V-gated cells (Fig. 3e) in OB-6 osteoblastic cells. Again, 4-OI pretreatment inhibited H_2_O_2_-induced apoptosis activation in OB-6 cells (Fig. 3d, e). Treatment with 4-OI alone failed to induce caspase and apoptosis activation in OB-6 cells (Fig. 3a–e).

In primary murine osteoblasts, 4-OI (25 μM) pretreatment attenuated H_2_O_2_-induced viability reduction (Fig. 3f). H_2_O_2_ provoked caspase-3 activation (Fig. 3g) and apoptosis induction (Fig. 3h, i). Apoptosis activation was evidenced by increased nuclear TUNEL staining (Fig. 3h) and an elevated number of Annexin V-gated cells (Fig. 3i). Significantly, 4-OI pretreatment largely attenuated H_2_O_2_-induced apoptosis activation in primary murine osteoblasts (Fig. 3g–i). Together, these results showed that 4-OI inhibited H_2_O_2_-induced cytotoxicity and apoptosis in osteoblasts.

### H_2_O_2_-induced programmed necrosis is inhibited by 4-OI in osteoblasts

**Fig. 4:**
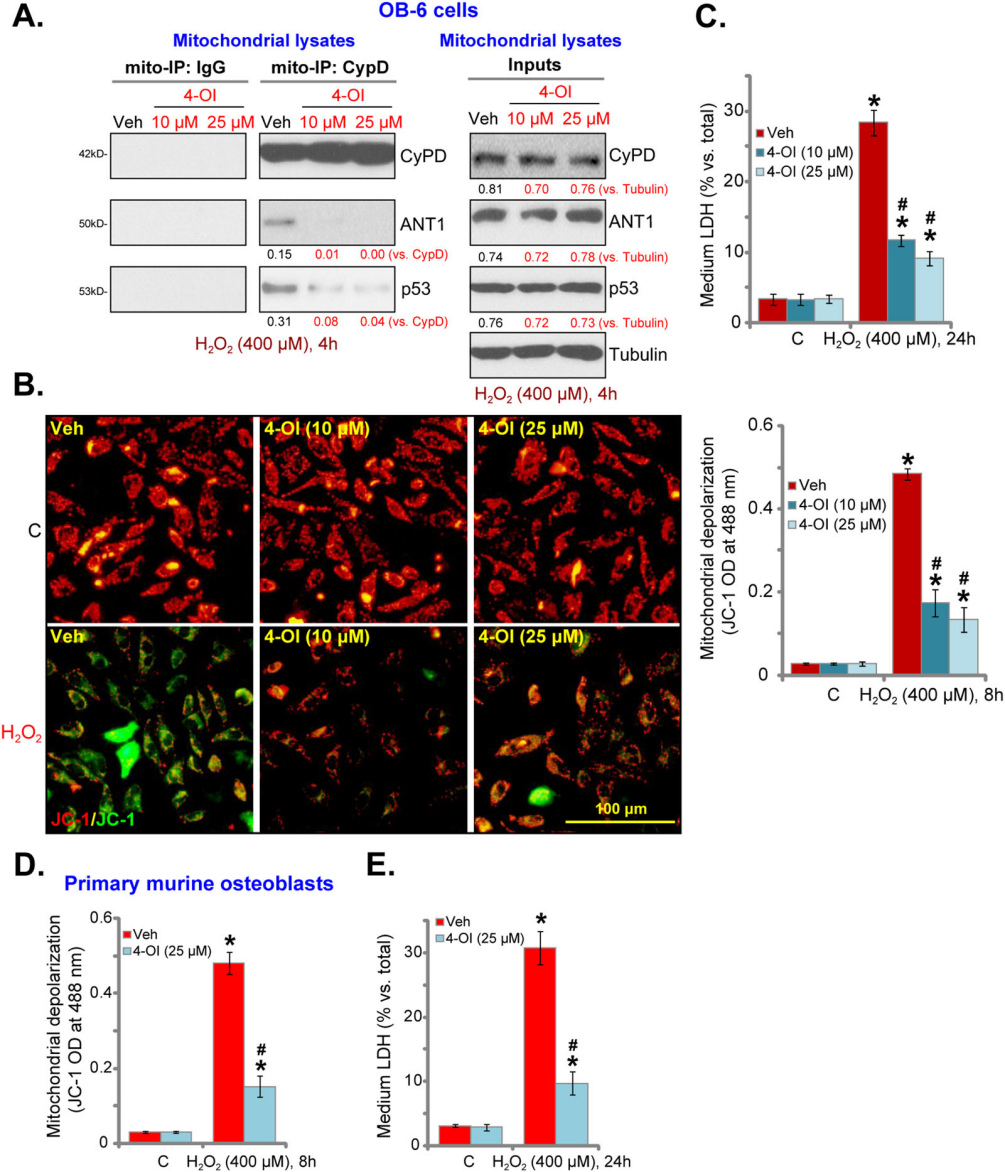
H_2_O_2_-induced programmed necrosis is inhibited by 4-OI in osteoblasts. Human osteoblastic OB-6 cells (**a**–**c**) or the primary murine osteoblasts (**d**, **e**) were pretreated for 2 h with 4-OI (10/25 μM) or vehicle control (“Veh”), followed by H_2_O_2_ (400 μM) stimulation. Cells were further cultured for applied time periods; mitochondrial CyPD-ANT1-p53 association and their expression are shown (**a**); mitochondrial depolarization was tested by JC-1 green monomer fluorescence (**b**, **d**), and cell necrosis examined by quantifying medium LDH release (**c**, **e**). Expression of the listed proteins was quantified and normalized to the loading control (**a**). Quantified values were mean ± standard deviation (SD, *n* = 5). “C” stands for the untreated control cells. *P < 0.05 vs. “C” cells. ^#^*P* < 0.05 vs. cells with H_2_O_2_ stimulation but “Veh” pretreatment. Experiments were repeated three times, with similar results obtained. Bar = 100 μm (**b**).

Besides apoptosis, H_2_O_2_ and oxidative stress could also induce programmed necrosis in osteoblasts^6,30,34,35^. Upon programmed necrosis, p53 would translocate to the mitochondria to form a complex with mPTP components cyclophilin D (CyPD) and adenine nucleotide translocase 1 (ANT1)^36–38^. This then leads to the opening of mitochondrial permeability transition pore (mPTP) and mitochondrial depolarization, and finally cell necrosis^36–38^. By applying the mito-immunoprecipitation (mito-IP) assay, we showed that CyPD immunoprecipitated with ANT1 and p53 in the mitochondria of H_2_O_2_-treated OB-6 osteoblastic cells (Fig. 4a). Tested by the mitochondrial accumulation of JC-1 green monomers, we also detected mitochondrial depolarization in these cells (Fig. 4b). Importantly, 4-OI (10/25 μM) pretreatment largely inhibited H_2_O_2_-induced CyPD-ANT1-p53 association (Fig. 4a) and mitochondrial depolarization (Fig. 4b) in OB-6 cells. Expressions of CyPD, ANT1 and p53 proteins in mitochondrial lysates were however unchanged (Fig. 4a, “inputs”).

Further studies confirmed that that H_2_O_2_ (400 μM) treatment induced OB-6 cell necrosis by causing significant LDH release to the culture medium (Fig. 4c), which was largely attenuated by 4-OI pretreatment (Fig. 4c). In the primary murine osteoblasts, mitochondrial depolarization (indicated by JC-1 green monomer fluorescence increase, Fig. 4d) and cell necrosis (medium LDH release, Fig. 4e) were detected following H_2_O_2_ stimulation. Again, such actions were largely inhibited by 4-OI (25 μM) pretreatment (Fig. 4d, e). These results indicated that 4-OI is able to inhibit H_2_O_2_-induced programmed necrosis in osteoblasts.

### Forced overexpression of IRG1 activates Nrf2 signaling and protects osteoblasts from H_2_O_2_

**Fig. 5:**
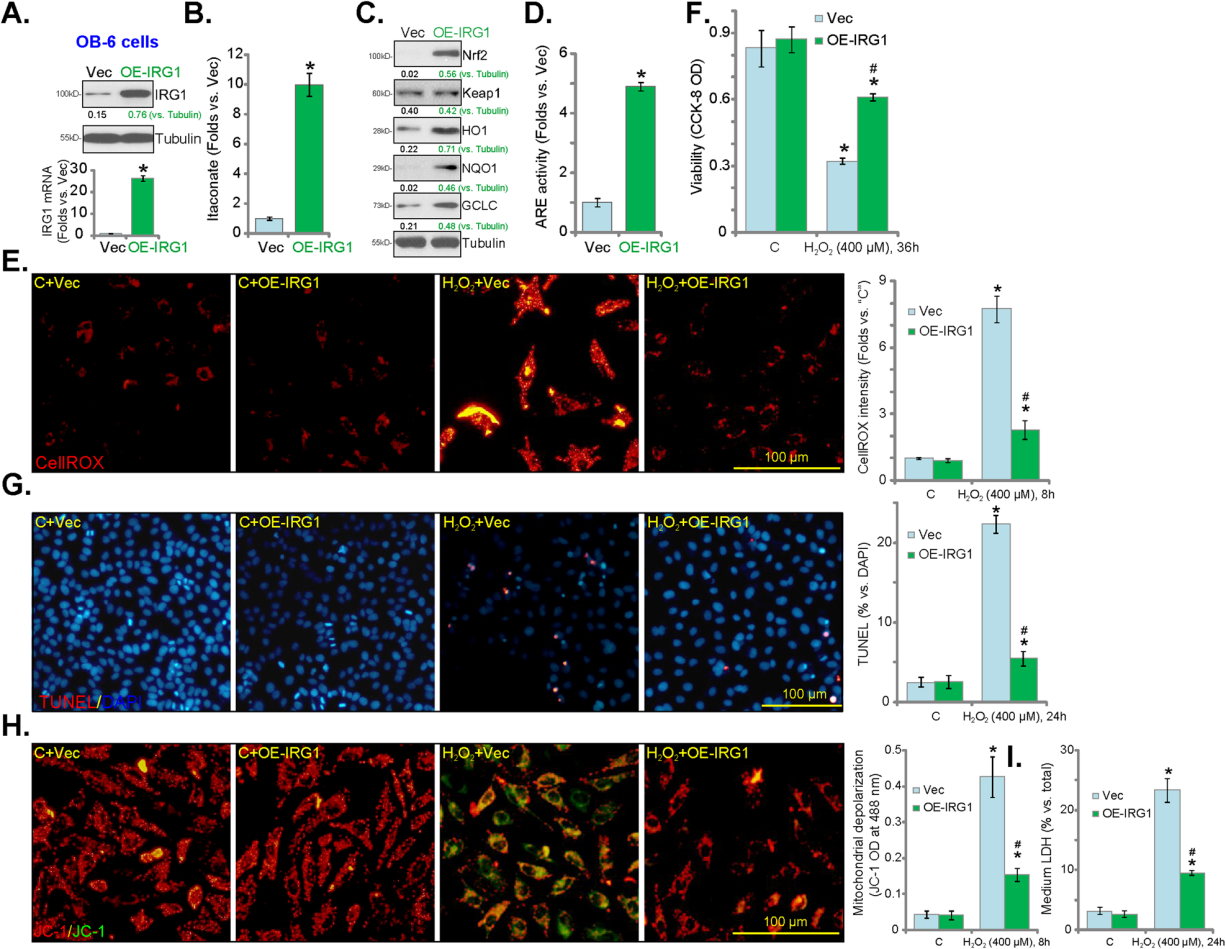
Forced overexpression of IRG1 activates nuclear factor E2-related factor (2Nrf2) signaling and protects osteoblasts from H_2_O_2_. Stable OB-6 cells with the lentiviral IRG1 expression construct (“OE-IRG1” cells) or the empty vector (“Vec” cells) were established, expression of listed genes was tested by qPCR and western blotting analyses (**a**, **c**). The itaconate contents (**b**) and relative ARE reporter luciferase activity (**d**) are shown. “OE-IRG1” cells or “Vec” control OB-6 cell were treated with H_2_O_2_ (400 μM) and cultured for applied time periods; reactive oxygen species (ROS) contents (CellROX intensity, **e**), cell viability (CCK-8 OD, **f**), cell apoptosis (nuclear TUNEL staining assay, **g**) were tested; Mitochondrial depolarization was tested by JC-1 green monomers (**h**), and cell necrosis examined by quantifying medium LDH release (**i**). Expression of the listed proteins was quantified and normalized to the loading control (**a**, **c**). Quantified values were mean ± standard deviation (SD, *n* = 5). “C” stands for the untreated control cells. *P < 0.05 vs. “C” cells. ^#^*P* < 0.05 vs. H_2_O_2_ stimulation in “Vec” cells. Experiments were repeated three times, with similar results obtained. Bar = 100 μm (**e**, **g**, **h**).

We next hypothesized that forced overexpression of IRG1, a rate-limiting enzyme in itaconate synthesis^14^, might increase endogenous itaconate production to activate Nrf2 cascade. A lentiviral IRG1 expression construct was transduced to OB-6 osteoblastic cells. Stable cells were established with FACS-mediated GFP sorting and puromycin selection: namely, OE-IRG1 cells. As shown, *IRG1* mRNA and protein levels were significantly increased in OE-IRG1 cells (vs. vector control cells, Fig. 5a). As a result, the cellular itaconate contents increased over tenfolds of vector control cells (Fig. 5b). Overexpression of IRG1 induced Nrf2 protein stabilization (Fig. 5c), and also increased the expression of HO1, NQO1, and GCLC proteins (Fig. 5c) in OB-6 cells. The relative ARE reporter luciferase activity was boosted, as well in the OE-IRG1 cells (Fig. 5d). Therefore, IRG1 overexpression increased itaconate production and activated Nrf2 signaling in OB-6 cells.

By analyzing CellROX fluorescence intensity, we demonstrated that H_2_O_2_-induced ROS production was largely inhibited in OE-IRG1 cells (Fig. 5e). Furthermore, H_2_O_2_-induced viability reduction was attenuated in IRG1-overexpressed OB-6 cells (Fig. 5f). TUNEL assay results showed that with IRG1 overexpression, H_2_O_2_-induced apoptosis activation was potently inhibited (Fig. 5g). Additional studies demonstrated that H_2_O_2_-induced mitochondrial depolarization (JC-1 green monomers accumulation, Fig. 5h) and cell necrosis (medium LDH release, Fig. 5i) were inhibited in OB-6 cells bearing IRG1 expression construct. These results showed that exogenous IRG1 overexpression attenuated H_2_O_2_-induced apoptosis and programmed necrosis in OB-6 cells.

### Nrf2 cascade activation mediates 4-OI-induced osteoblast cytoprotection against H_2_O_2_

**Fig. 6:**
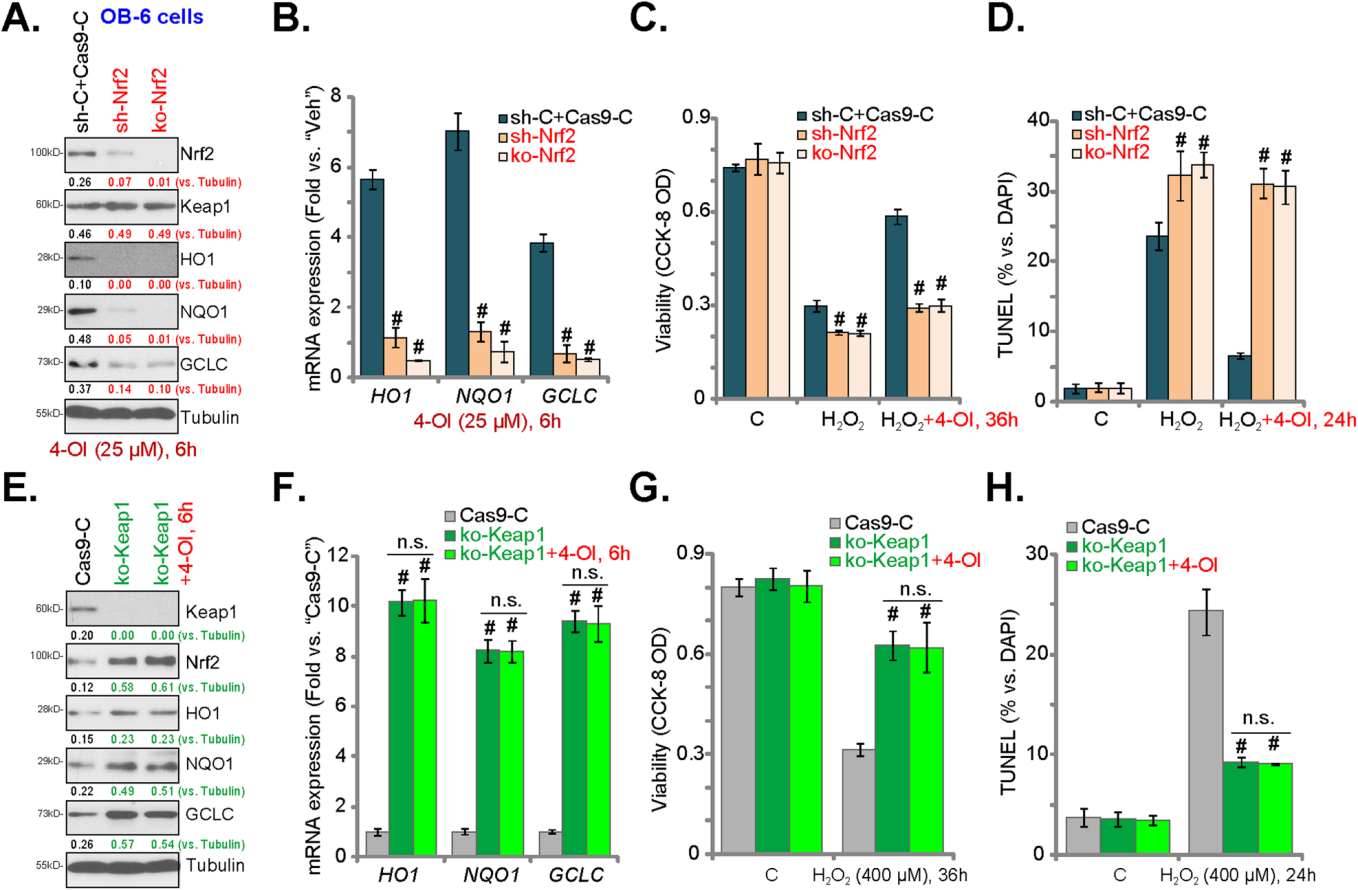
Nuclear factor E2-related factor 2 (Nrf2) cascade activation mediates 4-OI-induced osteoblast cytoprotection against H_2_O_2_. Stable OB-6 cells with Nrf2 shRNA (“sh-Nrf2” cells) or the CRISPR/Cas9-Nrf2-KO-GFP construct (“ko-Nrf2” cells), as well as the control OB-6 cells with scramble control shRNA plus CRISPR/Cas9 empty vector (“sh-C+Cas9-C” cells), were established and cultured, expressions of listed genes are shown (**a**, **b**). Cells were pretreated for 2 h with 4-OI (25 μM), followed by H_2_O_2_ (400 μM) stimulation, and cultured for applied time periods. Cell viability (**c**) and apoptosis (**d**) were tested by CCK-8 and nuclear TUNEL staining assays, respectively. Stable OB-6 cells with the CRISPR/Cas9-Keap1-KO-GFP construct (“ko-Keap1” cells) and the control OB-6 cells with CRISPR/Cas9 empty vector (“Cas9-C” cells) were established and cultured. Cells were treated with or without 4-OI (25 μM) for another 6 h, expression of listed genes is shown (**e**, **f**). These cells were pretreated for 2 h with 4-OI (25 μM), followed by H_2_O_2_ (400 μM) stimulation, and cultured for applied time periods. Cell viability (**g**) and apoptosis (**h**) were tested similarly. Expression of the listed proteins was quantified and normalized to the loading control (**a**, **e**). Quantified values were mean ± standard deviation (SD, *n* = 5). “C” stands for the untreated control cells. ^#^*P* < 0.05 vs. “sh-C+Cas9-C” cells or “Cas9-C” cells. “n.s.” stands for no significant difference (**f**–**h**). Experiments were repeated three times, with similar results obtained.

If 4-OI-induced osteoblast cytoprotection against H_2_O_2_ required Nrf2 cascade activation, it should be ineffective in Nrf2-depleted osteoblasts. To test this hypothesis, Nrf2 shRNA lentivirus was transfected into OB-6 cells, and stable cells (sh-Nrf2 cells) were established with puromycin selection. Alternatively, OB-6 cells were transduced with the CRISPR/Cas9-Nrf2-GFP-KO construct^18^. With FACS-mediated GFP sorting and puromycin selection, stable cells were established: namely, ko-Nrf2 cells. Control cells were transduced with scramble control shRNA and CRISPR/Cas9 empty vector: sh-C+Cas9-C cells. In sh-Nrf2 cells and ko-Nrf2 cells, 4-OI-induced Nrf2 protein stabilization (observed in sh-C+Cas9-C cells) was completely reversed (Fig. 6a). Additionally, mRNA and protein expressions of Nrf2-ARE-dependent genes (*HO1*, *NQO1*, and *GCLC*) by 4-OI were blocked with Nrf2 shRNA or KO (Fig. 6a, b). H_2_O_2_-induced cell death (CCK-8 OD reduction, Fig. 6c) and apoptosis (nuclear TUNEL staining, Fig. 6d) were intensified in sh-Nrf2 cells and ko-Nrf2 cells (vs. sh-C+Cas9-C cells). Importantly, 4-OI was unable to inhibit H_2_O_2_-induced cytotoxicity in Nrf2 silencing or KO OB-6 cells (Fig. 6c, d). Therefore, Nrf2 depletion reversed 4-OI-induced osteoblast cytoprotection against H_2_O_2_.

We further hypothesized that forced activation of Nrf2 should mimic 4-OI-induced osteoblast cytoprotection. A CRISPR/Cas9-Keap1-GFP-KO construct^18^ was transduced to OB-6 osteoblastic cells. Stable cells were again established with FACS sorting and puromycin selection: namely, ko-Keap1 cells. Keap1 KO induced significant Nrf2 cascade activation, led to Nrf2 protein accumulation (Fig. 6e), as well as increased expression of Nrf2-ARE-dependent genes (*HO1*, *NQO1*, and *GCLC*) (Fig. 6e, f). The ko-Keap1 cells were resistant to H_2_O_2_-induced cell death (Fig. 6g) and apoptosis (Fig. 6h). Importantly, in ko-Keap1 cells, 4-OI treatment failed to increase Nrf2 cascade activation (Fig. 6e, f). It did not offer further osteoblast cytoprotection against H_2_O_2_ (Fig. 6g, h). These results further supported that Nrf2 cascade activation mediated 4-OI-induced osteoblast cytoprotection against H_2_O_2_.

## Discussion

Activation of Nrf2 cascade can protect osteoblastic cells and primary osteoblasts from H_2_O_2_ and other oxidative stimuli^6,8,10^. Following activation, Nrf2 protein separates from Keap1, translocates to cell nuclei, and binds with ARE to promote transcription and mRNA expression of antioxidant and detoxifying genes, including *HO1*, *GSH*, *NQO1*, and *GCLC*^39–41^. These genes then exert significant ROS scavenging and antioxidant activities^9,42,43^.

Alkylating Keap1’s cysteine residues by itaconate can cause Nrf2-Keap1 departure and Nrf2 cascade activation^14,18^. Recent studies have developed 4-OI, the cell-permeable itaconate derivative, as a potent and efficient Nrf2 activator. Liu et al. showed that 4-OI activated Nrf2 cascade to protect neuronal cells from H_2_O_2_^18^. Activation of Keap1–Nrf2 signaling by 4-OI protected human umbilical vein endothelial cells from high glucose-induced oxidative injure and cell apoptosis as well^16^. In peripheral blood mononuclear cells of systemic lupus erythematosus patients, 4-OI activated Nrf2 signaling to inhibit pro-inflammatory cytokines production^17^.

Here in OB-6 cells and primary murine osteoblasts, 4-OI activated Nrf2 signaling cascade that led to Keap1–Nrf2 disassociation, Nrf2 protein stabilization, cytosol accumulation, and nuclear translocation. It also increased ARE reporter activity as well as mRNA and protein expression of ARE-dependent genes (*HO1*, *NQO1*, and *GCLC*). Functional studies showed that 4-OI pretreatment largely inhibited H_2_O_2_-induced ROS production and oxidative injury in both OB-6 osteoblastic cells and primary murine osteoblasts. Furthermore, H_2_O_2_-cell death and apoptosis in osteoblasts were largely attenuated with 4-OI pretreatment.

Existing studies have shown that mPTP is essential in mediating H_2_O_2_-induced cell death^44^. mPTP is formed by two structural components (ANT1 and VDAC, in the inner mitochondrial membrane) as well as the regulatory component CyPD (at the outer mitochondrial membrane)^36–38^. H_2_O_2_ stimulation would lead to p53 translocation to the mitochondria to form a complex with CyPD and ANT1, and cause mPTP opening, mitochondrial depolarization, and eventually cell necrosis^37,38^. The mitochondrial programmed necrosis pathway is also important for H_2_O_2_-induced osteoblast cell death^34^. Here, we showed that 4-OI inhibited H_2_O_2_-induced programmed necrosis by suppressing mitochondrial depolarization, mitochondrial CyPD-ANT1-p53 association, and medium LDH release in OB-6 cells and primary murine osteoblasts. This should explain the superior osteoblast cytoprotective action by the Nrf2 activator.

Activation of Nrf2 cascade using genetic strategies or pharmacological agents can efficiently protect osteoblasts from H_2_O_2_-induced oxidative injury and cytotoxicity^6,8,10^. The results of this study supported that the activation of Nrf2 is required for 4-OI-induced osteoblast cytoprotection against H_2_O_2_. In OB-6 cells, Nrf2 shRNA or CRISPR/Cas9-induced Nrf2 knockout blocked 4-OI-induced osteoblast cytoprotection against H_2_O_2_. Conversely, ectopic overexpression of IRG1 increased endogenous itaconate production and activated Nrf2 signaling cascade, while inhibiting H_2_O_2_-induced oxidative injury, cell death, and apoptosis. Similarly, forced Nrf2 cascade activation by CRISPR/Cas9-induced Keap1 KO mimicked 4-OI-induced actions and inhibited H_2_O_2_-cell death and apoptosis in OB-6 cells. Importantly, 4-OI failed to offer further osteoblast cytoprotection against H_2_O_2_ when Nrf2 was pre-activated in Keap1 KO cells. These results clearly demonstrated that Nrf2 cascade activation by 4-OI inhibited H_2_O_2_-induced oxidative injury and cell death in osteoblasts.

## Conclusion

Taken together, this study demonstrated that 4-OI activated Nrf2 signaling and protected osteoblasts/osteoblastic cells from H_2_O_2_-induced cytotoxicity. Activation of Nrf2 cascade by 4-OI could be a novel strategy to protect osteoblasts from oxidative injury.
